# Provider and female client economic costs of integrated sexual and reproductive health and HIV services in Zimbabwe

**DOI:** 10.1371/journal.pone.0291082

**Published:** 2024-02-12

**Authors:** Collin Mangenah, Euphemia L. Sibanda, Galven Maringwa, Justice Sithole, Stephano Gudukeya, Owen Mugurungi, Karin Hatzold, Fern Terris-Prestholt, Hendramoorthy Maheswaran, Harsha Thirumurthy, Frances M. Cowan

**Affiliations:** 1 Centre for Sexual Health and HIV/AIDS Research (CeSHHAR), Harare, Zimbabwe; 2 Department of International Public Health, Liverpool School of Tropical Medicine, Liverpool, United Kingdom; 3 Population Services International, Harare, Zimbabwe; 4 Ministry of Health and Childcare, Harare, Zimbabwe; 5 Population Services International, Washington DC, United States of America; 6 Policy, Advocacy and Knowledge Branch, UNAIDS, Geneva, Switzerland; 7 Institute of Global Health Innovation, Imperial College London, London, United Kingdom; 8 Department of Medical Ethics and Health Policy, University of Pennsylvania, Philadelphia, PA, United States of America; Clinton Health Access Initiative, SOUTH AFRICA

## Abstract

A retrospective facility-based costing study was undertaken to estimate the comparative cost per visit of five integrated sexual and reproductive health and HIV (human immuno-deficiency virus) services (provider perspective) within five clinic sites. These five clinics were part of four service delivery models: Non-governmental-organisation (NGO) directly managed model (Chitungwiza and New Africa House sites), NGO partner managed site (Mutare site), private-public-partnership (PPP) model (Chitungwiza Profam Clinic), and NGO directly managed outreach (operating from New Africa House site. In addition client cost exit interviews (client perspective) were conducted among 856 female clients exiting integrated services at three of the sites. Our costing approach involved first a facility bottom-up costing exercise (February to April 2015), conducted to quantify and value each resource input required to provide individual SRH and HIV services. Secondly overhead financial expenditures were allocated top-down from central office to sites and then respective integrated service based on pre-defined allocation factors derived from both the site facility observations and programme data for the prior 12 months. Costs were assessed in 2015 United States dollars (USD). Costs were assessed for HIV testing and counselling, screening and treatment of sexually transmitted infections, tuberculosis screening with smear microscopy, family planning, and cervical cancer screening and treatment employing visual inspection with acetic acid and cervicography and cryotherapy. Variability in costs per visit was evident across the models being highest for cervical cancer screening and cryotherapy (range: US$6.98—US$49.66). HIV testing and counselling showed least variability (range; US$10.96—US$16.28). In general the PPP model offered integrated services at the lowest unit costs whereas the partner managed site was highest. Significant client costs remain despite availability of integrated sexual and reproductive health and HIV services free of charge in our Zimbabwe study setting. Situating services closer to communities, incentives, transport reimbursements, reducing waiting times and co-location of sexual and reproductive health and HIV services may help minimise impact of client costs.

## Introduction

In Zimbabwe, the public health system is the largest provider of health-care services complemented by Church run Mission hospitals. However, despite growing demand, service provision has negatively been impacted by suppressed health-care budgets due to economic challenges over the last 2 decades. Non-governmental organizations (NGOs) have increasingly come in to support public health provision. Under the Zimbabwe Integrated Support Programme, Population Services International (PSI) worked with the Ministry of Health and Child Care to delay first birth among women, space their children at least 24 months apart and limit childbearing once their desired family size was achieved.

The initiative aimed to scale up access to a full range of modern SRH services including long-term reversible and permanent contraceptive methods and cervical cancer screening and cryotherapy. In addition to counseling to support informed choice, these services were delivered through innovative service delivery models which were already offering HIV testing services and limited short term contraceptive methods such as injectables, oral contraceptives, emergency contraceptives, male, and female condoms.

In Zimbabwe, the same unsafe sexual behaviours which predispose women and young girls to risk of HIV (human immuno-deficiency virus) infection also put them at increased risk of unplanned pregnancies, cervical cancer, unsafe abortions and sexually transmitted infections (STIs) such as syphilis, gonorrhea, and chlamydia [1]. Cervical cancer, strongly associated with HIV infection, is also the most prevalent cancer among Zimbabwean women with high mortality due to late screening [2–4]. A comprehensive package of high quality, effective, and integrated sexual and reproductive health (SRH) and HIV services is clearly required [5–10]. Integration involves offering clients both types of services during the same visit by one provider in the same room (“one-stop-shop/ kiosk” model) or during the same visit under the same roof or within the same clinic visit (“supermarket” model), with intra-referrals from one service to another [11–14].

Integrated SRH and HIV services may offer several benefits. Integration potentially enhances women and young girl’s access and utilisation of ordinarily separate SRH service components such as family planning (FP), STI screening and management, and cervical cancer screening and treatment in addition to HIV prevention, treatment, and care as well as tuberculosis (TB) screening and treatment services compared to stand-alone models [15–17]. Globally, integration of SRH and HIV/AIDS policies, programs, and services will help promote the achievement of international development goals and targets, such as the United Nations Sustainable Development Goal 3, which aims to ensure healthy lives and promote well-being of all [11].

A study in Uganda found integrated services promoted continuity of care, improved client-provider relationships, increased risk perception and HIV-SRH service demand and utilization among young people [8]. Moreover, HIV is transmitted predominantly sexually and vertically during childbirth and breastfeeding which are all reproductive health channels [18]. In Botswana, training and technical support provided to nine pilot sites between 2012 and 2013 as part of a countrywide scale up of SRH and HIV linkages with antiretroviral roll-out resulted in an 89% increase in female family planning clients accessing both HIV and family planning services. It also led to a 79% increase in the number of female clients at HIV service delivery points accessing both HIV and family planning services [9].

Integration of SRH and HIV services may be cost-effective as it reduces need for frequent health facility visits while potentially improving coverage of services [16, 19–22]. In addition to provider costs, client costs discourage health seeking behavior particularly among low-income earners and can impede continuity of treatment and care even where services are provided at no user fee. World Health Organization (WHO) and World Bank also estimate 100 million people in poorest countries are pushed into extreme poverty annually due to user fees or out-of-pocket expenses (OOPEs) when attending public health services [23–25].

Despite the evidence strongly suggesting integrated SRH and HIV services as an important entry point for expanded HIV testing and modern contraceptive use, the huge gaps between promising national integration policies and actual programme implementation, siloed funding by external partners, and service delivery approaches that are not client friendly are a major factor behind missed opportunities [26]. Also, integration can be associated with increased staff workload leading to long waiting times. It is therefore critical not only to assess how well integrated SRH and HIV services work, but also to evaluate the economic costs as there is a potential risk of introducing large, and potentially costly, yet ineffectual integration programmes at huge risk for health systems [27].

For Zimbabwe, which is rolling out integrated SRH and HIV services, it is necessary to understand both the provider costs and economic burdens borne by clients and households which could pose an impediment to provision (supply side) and access (demand side) to these services and to help optimize usage [21]. We undertook a cost analysis to evaluate the economic costs of integrated SRH and HIV services from both the provider and client perspectives in Zimbabwe.

## Materials and methods

### Cost study setting

We conducted a study to estimate the economic costs of four models of providing one-stop shop integrated SRH and HIV services to women in Zimbabwe as part of the *Programme of Research on the Integration of HIV and Sexual and Reproductive Health (SRH) Service*. Table 1 provides a detailed description of the service delivery models and the four sites purposively sampled for the costing study based on their advancement along the integration cascade. Cost data were collected at integrated non-governmental organisation (NGO) run clinic sites located in Harare, Zimbabwe’s capital city, Bulawayo, the second largest city and Mutare on the eastern border with Mozambique. The other NGO site was in Chitungwiza, a satellite town 30 km south of Harare.

**Table 1:** Models of providing one-stop shop integrated SRH and HIV services.

Site integration model type	NGO managed static model	Partner managed static model	Private Public Partnership (PPP) static clinic	PSI Zimbabwe managed mobile outreach^1^
**Service managed by**	PSI Zimbabwe	Local Authority	MoHCC^2^	PSI Zimbabwe
**Funder**	USAID^3^	USAID	USAID	USAID
**Location**	Chitungwiza	Mutare	Chitungwiza	Harare
**Rural v Urban**	Urban	Urban	Urban	Rural
**Opening time/ Operations details**	8 am to 5pm	8 am to 5pm	8 am to 5pm	8 am to 5pm
**User fees**	Optional	Optional	Optional	Optional
**Coverage**	PSI Zimbabwe. Every province of the country. >500000 people annually	PSI Zimbabwe in partnership. Every province of the country, reaches PLHIV^4^	Private, public or NGO providers, city health clinics^5^. FP services for 73.4% of women.	PSI Zimbabwe. All 62 rural districts of Zimbabwe. Rural poor, marginalised communities, and at-risk populations.
**HTC services offered prior to integration**	HTC^6^, TB^7^ screening with smear microscopy and GeneXpert testing, CD4^8^ cell count, & post-test support services for PLHIV.	HTC, TB screening with smear microscopy and GeneXpert testing, CD4 cell count, & post-test support services for PLHIV.	Sites offering maternity, delivery, MNCH^9^ & PMTCT^10^.	HTC, TB screening with smear microscopy and GeneXpert testing, CD4 cell count, & post-test support services for PLHIV.
**Services available in addition to HIV care**	Limited FP^11^ e.g., LARM’s^12^ limited to implants available in select sites, STI^13^, cervical cancer screening & cryotherapy	Limited FP e.g., LARM’s limited to implants available in select sites, STI, cervical cancer screening & cryotherapy	High unmet need for FP. Inadequate package of SRHR^14^ & HIV services. Cervical cancer screening not offered.	Limited FP e.g., offer only short-acting FP services, STI, Female and male sterilisation, cervical cancer screening & cryotherapy
**Additional Integrated services**	Co-brand with new SRHR franchise brand. Addition of LARM’s e.g., IUDs^15^, implants. Expand services from 10 to 20. Screening & treatment for STIs, cervical cancer screening, referral to public sector sites for appropriate follow-up. Comprehensive services for survivors of sexual violence	Co-brand with new SRHR Integrated service package. Addition of LARM’s e.g., IUDs, implants. Screening & treatment for STIs, cervical cancer screening, referral to public sector sites for appropriate follow-up. Comprehensive services for survivors of sexual violence	65 new clinics, Partial franchise model for SRHR service package provision. LARM’s e.g., IUDs, implants. STI screening & treatment, cervical cancer screening, referral to public sector sites for appropriate follow-up. Comprehensive services for survivors of sexual violence	Co-brand outreach teams with new SRHR Integrated service package. Addition of IUDs, implants. Female and male sterilisation, STI screening and treatment and cervical cancer screening and cryotherapy.

^1^ Population Services International

^2^ Ministry of Health and Child Care

^3^United States Agency for International Development

^4^ People Living with HIV

^5^ Non-Governmental Organisation

^6^ HIV Testing and Counselling

^7^ Tuberculosis

^8^ Clusters of Differentiation 4

^9^Maternal, New born and Child Health

^10^ Prevention of Mother to Child Transmission

^11^Family Planning

^12^ Long Acting Reversible Methods

^13^ Sexually Transmitted Infections

^14^ Sexual and Reproductive Health Rights

^15^ Intrauterine Device

Ethical approval for the Programme of Research on the Integration of HIV and Sexual and Reproductive Health Services was provided by the Medical Research Council of Zimbabwe (MRCZ) and the University College London (UCL) Ethics Committee. Only verbal consent was administered following exemption from written informed consent by the institutional review board of the MRCZ as that would typically take at least 15 minutes for a 5-minute anonymous questionnaire.

We assessed the relative cost per visit of five integrated SRH and HIV services. Costs were assessed for HIV testing and counselling (HTC), STI screening and treatment, tuberculosis screening (TB) with smear microscopy, family planning (FP), and cervical cancer screening and treatment employing visual inspection with acetic acid and cervicography (VIAC) and cryotherapy. The Chitungwiza, New Africa House, and Bulawayo sites are directly run integrated SRH and HIV fixed NGO sites. The Mutare site is an NGO partner managed (Mutare City Council) fixed site. The Private Public Partnership (PPP) Profam clinic site was in a public health facility, Chitungwiza Hospital. For the integrated mobile outreach model, we assessed costs based on activities of outreach teams from New Africa House NGO site in Harare.

### Costing overview

This economic cost analysis adopted both the provider and client perspective, including both costs of delivering integrated SRH and HIV services and costs borne by clients (transport, absenteeism from work, and caregiver costs) accessing services [28]. Our costing approach followed international costing guidelines [28, 30]. The costing involved first a facility bottom-up costing exercise (February to April 2015) conducted to quantify and value each resource input consumed for provision of individual SRH and HIV services (S1 File). Secondly overhead financial expenditures were allocated top-down from central office to sites and then respective integrated service based on pre-defined allocation factors derived from both the site facility observations and programme data for the 12 month period January to December 2014 [28–32].

### Provider cost data collection

At each facility SRH and HIV service utilization data were collected for the retrospective 12-month period (January to December 2014) from program records, monthly reports, registers maintained in each unit or service department. This service utilisation data was important as it is directly related to the quantities of specific inputs that feed into individual integrated SRH and HIV services (S1 Table). We worked with the provider monitoring and evaluation (M&E) teams to ensure that site records matched central databases on which cleaned and verified data was stored and to ensure data were de-duplicated.

We conducted time and motion analysis to understand how health provider staff shared their time across departments and the specific duration of tasks in integrated service provision to individual clients. Time and motion analysis is the gold standard for measuring staff allocation of time through direct observation [31–33]. Health provider staff were randomly selected from a departmental staff roster or list of those providing integrated services (we aimed for all or every second participant if more than six) and asked to provide written informed consent to be observed during their work. Trained economics data collectors (two) conducted observations of health providers while they were providing integrated services, recording how much time it took to conduct specific activities. Observations were conducted from outside of consultation rooms to ensure client confidentiality was maintained. We then used the mean time estimates from this process not only to directly estimate provider time costs per individual service but also as an allocation factor for overhead personnel (supervision and other support personnel time) costs.

### Client costing overview

We collected costs borne by women at the static NGO sites in Harare, Chitungwiza, and Bulawayo (S2 File). Client costs data collection among women attending the mobile outreach clinic model was precluded by study budget constraints. Female clients (n = 856) who had received one or more of the integrated services (SRH and HIV) were subsequently asked to participate in a 5-minute exit interview conducted by trained research assistants using a structured questionnaire. Costs accessed included direct service plus non-service costs and opportunity costs of time incurred by women seeking integrated SRH and HIV services [33]. Direct non-service costs included lost income/productivity/wages from absenteeism whilst seeking health services, transport costs incurred travelling to and from health facilities, caregivers’ time and transportation costs, food, and other incidentals.

Clients were asked to indicate occupation, related earnings in US$, transport cost to and from the clinic facility, time taken to get to the clinic (we assumed it would take equal time to get back home), time spent at the clinic, any additional money spent on food and other expenses, or any service (user fees) payments made. Clients were also asked to provide details of any accompanying caregivers on the day, their occupation, earnings per month in US$ and any costs incurred by caregivers while accompanying the women to the clinic facility. Other data collected was on patient characteristics, service(s) sought and recommended by staff and any family planning methods offered and taken up. Costs associated with seeking integrated services were recorded and uploaded in real time using tablets.

### Cost data analysis

#### Provider costs

Provider costs were captured and analysed in a Microsoft Excel spreadsheet that we specifically designed to record and estimate the economic costs of providing integrated services. Each resource input identified from the bottom-up costing exercise and required to provide individual integrated SRH, and HIV services was valued using prices from the NGO’s finance department and the NATPHARM reference pricing list [34]. Overhead financial expenditures were allocated step wise from central office to sites and integrated SRH and HIV services [35, 36]. Shared overhead costs such as management, vehicles and space were allocated to clinic services based on recorded usage. Site security, reception, and caretaker services as well as utilities were allocated based on space utilised by respective integrated SRH and HIV services. S1, S6 and S7 Tables provide details on integrated SRH and HIV service utilisation, workforce composition per site and department and space measurements which we used to allocate shared costs. Capital and recurrent costs for each integrated SRH and HIV service were estimated separately and then added up to derive a full total service cost. Unit costs per client visit were estimated by dividing the full total service cost by the number of clients seen per service [33, 37]. All costs were analysed in 2015 United States dollars. United States dollars were the principal currency in use in Zimbabwe at the time following the demise of the local currency earlier in 2009 due to hyper-inflation [38].

#### Client costs

Time taken off by individuals from their daily work to seek health services potentially represents a loss not only to themselves directly in the form of lost income, but also to the economy overall. In this analysis we assessed the opportunity costs incurred by women (productivity losses) and their caregivers seeking integrated SRH and HIV services. The human capital approach, the traditional method for estimating productivity losses, assumes that individuals have the potential to produce a stream of outputs (productivity) over their working life and measures lost productivity as the amount of time by which working life is reduced due to illness [39]. This work time lost is then valued at the market wage which reflects the value of that work to society. We multiplied the average time spent seeking integrated services (including travel to facility, time spent at facility and anticipated time travelling back home) by clients self-reported earnings per hour. Although traditionally, analysis of lost productivity has focused on paid work, there is increasing recognition that people’s unpaid productivity, through roles such as caring for children or relatives, household tasks, and volunteering, also makes important contributions to society [40, 41]. In our analysis therefore to account for any lost productivity for clients and caregivers who report no earnings due to being unemployed, students or other we impute the median of the stipulated monthly minimum wages across 28 Zimbabwean industrial sectors ($120/month) as a proxy for (S8 Table) lost income [42, 43]. In univariate and multivariate sensitivity and scenario analysis we alternatively impute the median monthly earnings of the clients and accompanying caregivers who reported being employed ($200 instead of $120/month) to those reporting no earnings.

## Results

### Integrated service utilisation

S1 Table presents overall utilisation data by site for all integrated SRH and HIV services during the corresponding 12 months. Utilisation data is based on the pre-existing NGO provider M&E records. For the outreach service 77,278 clients accessed services composed of 69,942 (91%) for HIV testing and 7,063 (9%) for FP. Of the total 49,607 clients accessing services at the New Africa House (NAH) site 41,177 (83%) accessed HIV testing, 3,354 (7%) FP, 2,958 (6%) cervical cancer screening and cryotherapy, 1,561 (3%) TB screening. 17,934 clients visited the Mutare site with 12,005 (67%) coming for HIV testing, 3,881 (22%) for FP and 1,922 (11%) for FP. The PPP site at Chitungwiza hospital had a total of 6, 037 visits broken down into 5,042 (84%) for FP, 412 (7%) HIV testing, 327 (5%) STI screening and treatment and 256 (4%) for cervical cancer screening and cryotherapy.

### Client characteristics

In total 856 female clients were recruited for exit interviews during the period February to April 2015. We aimed to recruit every third participating woman who had just completed receiving (exiting) any (or all) of the integrated services. However, due to time and study budget constraints our approach also allowed a more purposive sampling strategy when the client flow was slower. S2 Table presents main reason for visit for the sample drawn from New Africa House (n = 456), Chitungwiza (n = 200), and Bambanani (n = 200). Most clients attended integrated clinic facilities to access FP services (48%), followed by cervical cancer screening and treatment (22%), and HIV testing (16%). At Chitungwiza New Start Centre (NSC) however, clients mainly attended for cervical cancer screening (42%). 42%, 27% and 19% of clients reported seeking 1, 2 or 3 other services in addition to their main reason for visiting on the day. The other 12% of clients reported seeking 4, 5 or 6 other services in addition to their main reason for visiting.

S3 Table presents HIV status, presence of caregiver, client and caregiver employment status, and mode of transport used to travel to the health facility. A total of 595 (70%) clients self-reported testing HIV negative, 243 (28%) HIV positive, 4 (1%) preferred not to say, and 14 (2%) didn’t know. For employment status 318 (37%) clients interviewed were unemployed, 154 (18%) reported performing unskilled labour, and 103 (12%) semi-skilled. Professionals numbered 79 (9%), students 30 (4%) and 172 (20%) reported as other. Clients accompanied by a caregiver to the clinic numbered 151 (17%). Of these caregivers 59 (45%) were unemployed, 15 (12%) unskilled, 13 (10%) semi-skilled, and the rest professionals (22, 17%), students (8, 6%), and other (13, 10%).

S4 Table summarizes time spent by clients accessing the integrated SRH and HIV services and shows clients took an almost similar amount of time seeking integrated SRH, and HIV services across the three clinic facilities (Mean = 5 hours). Time (both waiting and receiving services) at the facility (Mean = 3 hours) contributed more to income/productivity losses (63%) in comparison to travel time to (18%), and from clinics (18%), respectively.

### Unit costs per service visit by integration model

S5 Table presents the total program and unit cost per visit for each of the 5 integrated SRH and HIV model sites. Total programme costs per model site were $466,000 for Chitungwiza NGO site, $288,000 for Mutare NGO site, $723,000 for the larger Harare NGO site, and $1,168,000 for NGO outreach respectively. The total program cost for the Chitungwiza Profam clinic located in a government facility was $101,000.

#### Mean costs per service

**Fig 1:**
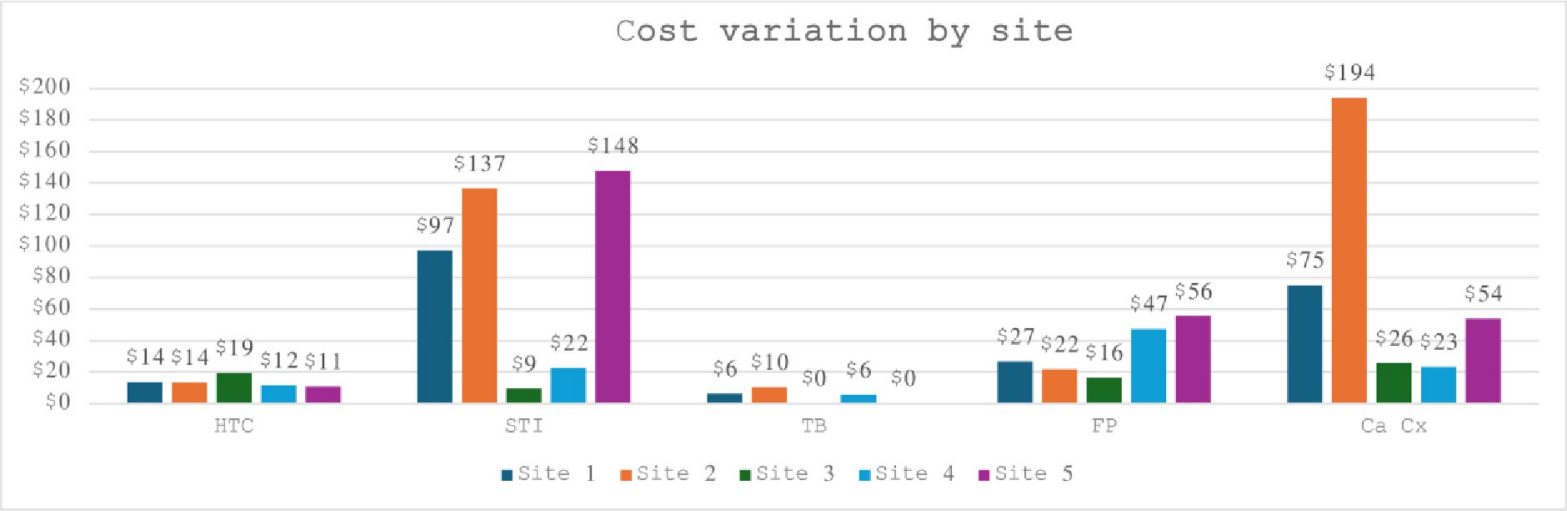
Costs of the integrated SRH and HIV services (2015 US dollars).

The mean cost per visit for the 5 integrated SRH and HIV services ranged from a low of $6.06 (TB screening and treatment visit) to a high of US$194 (cervical cancer screening and cryotherapy) at the partner managed model site. Results show some variability in costs per visit across the model sites with highest variability observed for cervical cancer screening and cryotherapy which ranged between US$23 at the NGO managed site and US$194 for the NGO partner managed site. HTC showed least variability in costs ranging between US$11 for the NGO mobile outreach and US$19 at the PPP model site (Fig 1).

**Fig 2:**
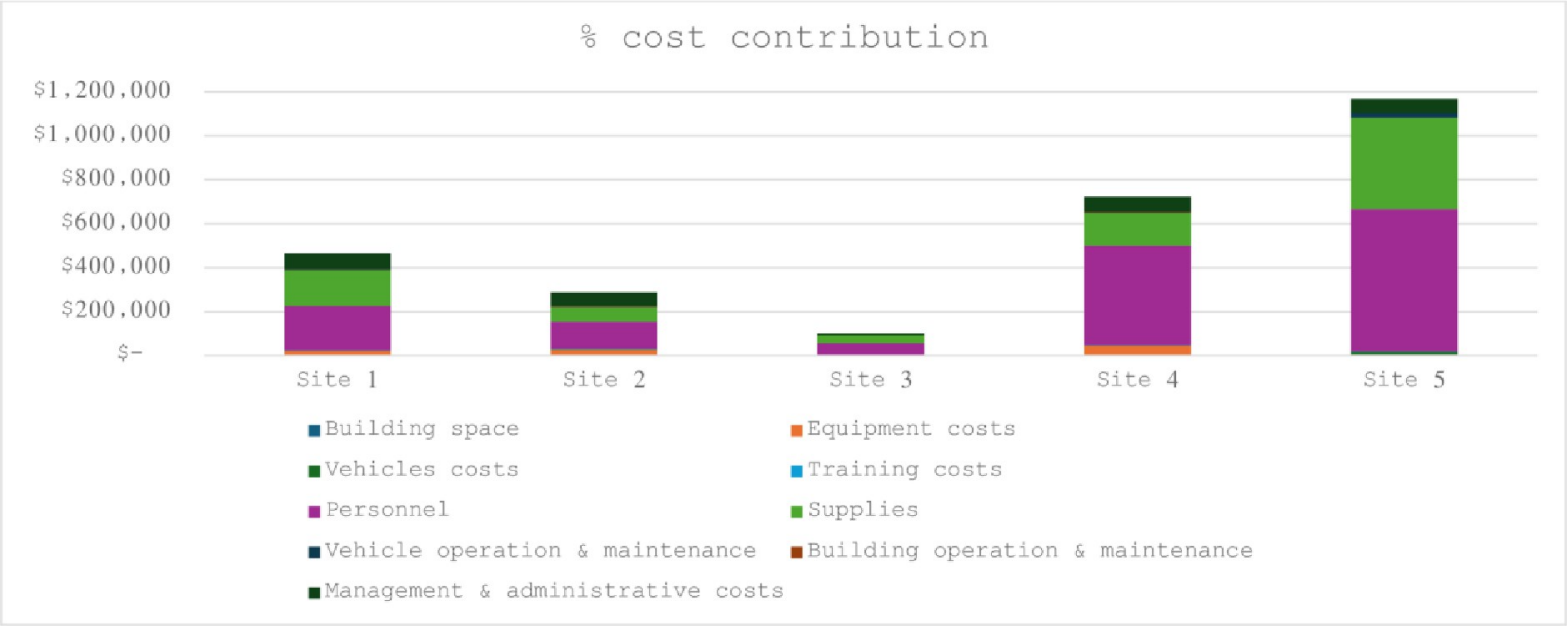
Total cost contribution.

The model with the lowest unit costs per visit overall was the NGO outreach model site (US$15) and the highest in the PPP model ($17). The model site that offered integrated services at the lowest unit costs was PPP (US$19 for HTC, US$9 for STI screening, $16 for FP and US$26 for cervical cancer screening and cryotherapy) although costs for this site exclude TB screening costs. The model site with the highest costs was partner managed site (US$14 for HTC, US$137 for STI screening, $10 for TB screening, US$22 for FP and US$26 for cervical cancer screening and cryotherapy). Unit costs were mainly recurrent costs driven (Fig 2) particularly by personnel 54% (42% - 62%), supplies 30% (23% - 36%) and management and administration costs 10% (5% - 22%). In sensitivity and scenario analysis we varied the key cost contributors, personnel, supplies and management and administration up and down (+/- 20%) to assess impact of future salary adjustments, inflation and clinic level client throughput. Results remained robust ranging from 53% to 54% for personnel, 30% to 31% for supplies and unchanging at 10% for management and administration.

### Client costs

All clients in this study sample incurred zero OOPE’s for services as these were fully borne by the provider. However, clients incurred other direct non-service costs of transport (mean = $1.67) to and from the facility, food, and other related expenses (mean = $1.16), and lost income (productivity losses) due to time spent seeking services (mean = $7.27). For a breakdown of the component costs see Table 2.

**Table 2:** Client costs associated with accessing integrated SRH, and HIV services.

Variables	Bambanani	Chitungwiza	NAH	Overall
** **				
No of exit interview clients	200	200	456	856
Average total annual family income (US$)	$4,663	$5,616	$5,151	$5,143.33
** Average annual client income (US$)**	$1,925	$2,616	$2,447	$2,329.26
** Average annual caregiver income (US$)**	$2,738	$3,000	$2,704	$2,814
**Average earnings per day ($) [average annual family income/228]**	$20.45	$24.63	$22.59	$22.56
**Average earnings per day ($) [average annual client income/228]**	$8.44	$11.47	$10.73	$10.22
**Average earnings per hour ($) [client earnings per day/6.5 hour day]**	$1.30	$1.77	$1.65	$1.57
Client cost (US$)	$7.96	$13.03	$9.21	$10.10
Direct service costs (US$)	$0.00	$0.00	$0.00	$0.00
Direct non-service costs (US$)	$7.96	$13.03	$9.21	$10.10
Average total transportation costs (US$)	$1.21	$1.50	$1.95	$1.67
***Average transportation cost to clinic (US$)***	$0.63	$0.73	$1.00	$0.85
***Average transportation cost from clinic (US$)***	$0.58	$0.77	$0.95	$0.82
Average food and other incidental costs (US$)	$1.17	$1.20	$1.11	$1.16
Opportunity cost of time (US$) [Average time spent x average earnings per hour]	$5.58	$10.33	$6.15	$7.27
***Average time spent travelling to facility (in mins)***	*<1* (<1–3)	*<1* (<1–5)	*<1* (<1–6)	*<1* (<1–6)
***Average time spent at facility (in mins)***	3 (2–8)	*4* (<1–8)	2 (1–8)	3 (<1–8)
***Average time spent travelling home (in mins)***	*<1* (<1–3)	*<1* (<1–5)	*<1* (<1–6)	*<1* (<1–6)
Total time spent accessing integrated services (Hours)	4	6	4	5
Client cost/daily family income (%)	39%	53%	41%	45%
Client cost/daily client income (%)	94%	114%	86%	99%

The main client cost driver was lost income (productivity losses) from absenteeism measured in time spent travelling (to and from facility) and at the clinic facility waiting and seeking integrated SRH and HIV services (S4 Table). The longer the distance (measured through time spent travelling to and from facilities), the higher the cost of time expended by clients accessing integrated SRH, and HIV services. Its proportional contribution to client cost per visit was 72% compared to 17% for transport, and 11% for other incidental expenses. Overall, mean total client cost per visit was $10.10, a figure which accounted for 45% of the daily family income ($22.56). When alternatively imputing the median monthly earnings of only the clients and accompanying caregivers who reported being employed ($200 instead of the base case $120/month) to those reporting no earnings client costs increased from $10.10 to $11.62 (56% of the daily family income).

**Fig 3:**
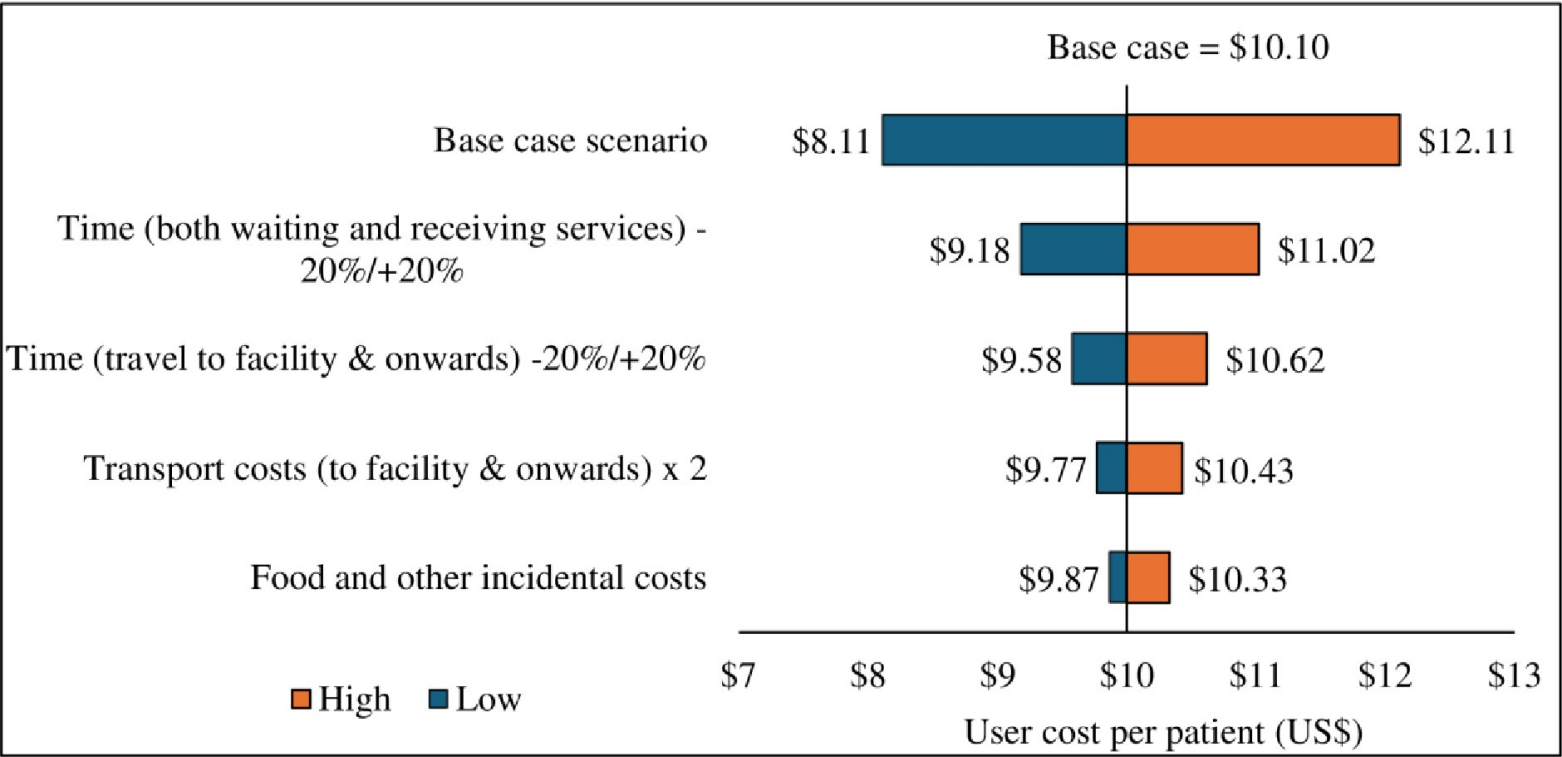
Sensitivity and scenario analysis.

In univariate and multivariate sensitivity and scenario analysis (Fig 3 above) client costs were highly sensitive to variations in time travelling to the facility, receiving services and onwards to next destination.

## Discussion

This to our knowledge is the first study to evaluate both provider and client costs of integrated SRH and HIV services across various service delivery models in Zimbabwe. It potentially contributes to a better understanding of the funding needs required to ensure well-funded, managed and delivered health services as called for in “The Framework on Integrated People-Centred Health Services” which aims to support countries progress towards universal health coverage by shifting away from health systems designed around diseases and health institutions towards health systems designed for people [44]. It provides comprehensive evidence on service utilisation, costs of five of the integrated SRH and HIV services, a complete breakdown of the various cost components contributing to total costs of the five services as well as the main cost contributors to client costs. Services with higher utilisation rates were found at sites that had been offering those services for longer (before launch of integrated services) pointing to service maturity.

Personnel, supplies and management and administration costs were the main cost drivers highlighting the relative influence of fixed inputs when uptake of services across sites is lower or higher. Variation in unit costs of integrated SRH and HIV services (partly explained by variation in the proportion of visits) and percentage distribution of cost components across the four models suggests room to improve efficiencies. Elsewhere demand generation initiatives have been shown to increase demand for other integrated HIV programs such as VMMC leading to lower costs per client [45, 46]. Results of this study highlight the need for more demand generation for integrated SRH and HIV services to fully utilise fixed capital inputs such as human resources and thus achieve lower unit costs.

The study shows that provision of integrated SRH and HIV services in Zimbabwe is generally not only inexpensive, and feasible but it is also cheapest within a PPP Profam model integrated into public sector facilities. In Kenya and Swaziland variability in unit costs and cost components suggested potentially reduced costs through better use of both human and capital resources [45, 46]. Staff salaries made up a significant proportion of total costs across all services followed by other costs such as diagnostics and supplies. Furthermore there was wide variation in unit cost per visit for all services across facilities. The least variation was found in family planning services and the widest in HIV care [45, 46].

We also examined the direct and indirect economic costs incurred by clients accessing integrated SRH and HIV services. For this study, although clients incurred zero direct payments for integrated SRH, and HIV services (fully absorbed by the provider as is often the case in NGO and public sector service for no fee facilities); clients did incur other non-service costs, including transport, food, and productivity losses. Non-service costs constituted 86% of the daily family income and were more than double the daily client income clearly presenting a significant barrier to integrated service access. Furthermore, attendees from poorer households (as measured through earnings) parted with a higher proportion of their daily income in-order to access same services compared to those with higher daily incomes buttressing findings from elsewhere that show client costs discourage access to public health services. Client costs of accessing integrated SRH and HIV services virtually wiped out the daily client income and were 45% of the daily family income. Poorer households therefore lost a higher proportion of their daily income accessing integrated SRH and HIV services in comparison to those with relatively higher daily income.

Our findings suggest that the main client cost driver was lost income (productivity losses) resulting from absenteeism measured in time at clinic facility seeking services, and distance (measured through time spent travelling to and from facilities). The higher the amount of time spent at facilities by clients and the further away from the facility a client’s home was situated, the higher the productivity losses. In Malawi living far from public health facilities, and high transportation charges were found to be barriers to care when ART scale up was rolled out [47, 48].

According to the World Bank, World Health Organisation, and others the rural poor have been shown to sell off prized assets such as cattle, goats, and farming implements just to afford OOP payments for transport, and medical care in addition to absenting themselves from their daily sources of income [49–52]. This however applies mainly in-order to access emergency medical care rather than for routine clinic attendance. Another study in Kenya concluded that most of the households pushed and trapped into poverty due to medical expenses were those from rural areas as poverty levels differed between urban, and rural areas [53]. Studies in Malawi show that even in the absence of direct service payments, client OOPEs were still considerable and imposed debilitating financial burdens on households and clients [33] Additionally, it was the poorest that were hit hardest and who were plunged further into poverty [48].

Our study has some limitations. Firstly, due to time and study budget constraints we used a purposive sampling strategy to identify clinic facilities in which to conduct the cost study based on advancement along the integration cascade. Again due to time constraints individual clients were also recruited for our exit interviews using purposive sampling limiting representativeness of our sample. Results may therefore not be fully representative particularly of other facilities and clients seeking integrated services in the same or similar facilities in Zimbabwe, or elsewhere. Furthermore, our study was conducted within the two largest cities (Harare, and Bulawayo), as well as Chitungwiza and Mutare which are also home to relatively large populations in Zimbabwe. Client cost results from these settings may not be fully representative of clients receiving integrated services in other parts of the country particularly in smaller towns, rural, mining and farming communities.

In addition, the client cost study relied on the relatively low cost self-reported data methodology which has been shown subject to social desirability bias and prone to either deliberate or erroneous under or over-estimation of income, transport, food and other cost estimates especially when there is an expectation of reimbursement [54, 55]. In other settings, to reduce reporting bias, the opportunity costs of time spent seeking healthcare services have been based on direct observation through time and motion studies which have included direct measurement of the time taken to travel to and from facilities as well as identifying common types of transport and how much they cost.

Despite these limitations however our study approach has some important strengths to note. Firstly we aimed for a mixed methods approach to balance the delicate trade-off between bottom-up accuracy at tracking and assigning resource use at the site and service level and top-down simplicity which is less accurate and obtains more valid cost estimates [56, 57]. The choice of costing method has been shown to significantly change results of economic evaluations [58, 59]. Whereas bottom-up micro costing is considered to underestimate overheads, top-down financial expenditure analysis tends to underestimate site level economic costs [36]. In addition, as seen elsewhere in the region, overreliance on bottom‐up ingredients costing has been shown to create substantial budgetary pressures because real expenditure (as captured in top‐down methods) exceeds the estimates thereof [59]. Secondly our study takes a societal perspective which more fully accounts for the opportunity costs borne by society compared to the narrower provider approach which does not consider alternative resource uses outside the public health care sector, which potentially yield greater welfare to society [60].

## Conclusion

As shown above, and despite the limitations, the results of our cost analysis have clear implications for enhanced integrated SRH and HIV delivery outcomes in Zimbabwe, and other sub-Saharan Africa (SSA) jurisdictions. Given international recommendations for integration of SRH and HIV services from a public health perspective, results from our Zimbabwe study provide further evidence showing that apart from being feasible, integration is a more efficient way of reaching women and young girls who have been shown to be more vulnerable to HIV infection in high HIV prevalence settings such as SSA in general and Zimbabwe in particular. Demand generation for integrated SRH and HIV services can help achieve lower unit costs. Where possible, integrated SRH and HIV service providers in Zimbabwe should negotiate lower drugs, diagnostics, and supplies prices as these were also an important contributor to unit costs of services. It is, however, important to note that other settings such as farming, and mining areas may require a balance between seeking more efficient models with other important service delivery objectives such as accessibility of services in the first place.

In this study, the direct non-service costs of seeking integrated SRH, and HIV services have been shown to be high for clients in urban Zimbabwe despite being available free of charge. To mitigate these effects providers of integrated SRH and HIV services in Zimbabwe, and elsewhere need to minimise impact of client costs by reducing times (both waiting and receiving services), providing transport reimbursements, opening clinics outside working hours and situating services closer to communities/workplaces in-order to compensate for the negative effect of OOPE’s. Potentially high productivity losses, transport, and other costs will likely result in a reluctance to utilise integrated SRH and HIV services. In an informal economy such as Zimbabwe’s the effects of a day away from one’s place of work are more acute for those running informal businesses, most of which are owner operated, in comparison to those who are formally employed and can afford to take a day off from work to access integrated SRH and HIV services.

## Supporting information

S1 Table Integrated SRH and HIV service utilisation data. (DOCX)

S2 Table Main reasons for health facility visit. (DOCX)

S3 Table Characteristics of clients and their caregivers. (DOCX)

S4 Table Breakdown of time spent accessing integrated SRH and HIV services. (DOCX)

S5 Table Costs of integrated SRH and HIV services for several model sites (2015 US dollars). (DOCX)

S6 Table Workforce composition per site and department. (DOCX)

S7 Table Space measurements per site. (DOCX)

S8 Table Minimum wage in Zimbabwe for the mining, printing and packaging, ceramic, insurance, transport industry. (DOCX)

S1 File Costing overview. (DOCX)

S2 File SRH & HIV integration client questionnaire. (DOCX)
