# Supplementary material for: Provider and female client economic costs of integrated sexual and reproductive health and HIV services in Zimbabwe
Source: PLoS One. 2024 Feb 12;19(2):e0291082. doi: 10.1371/journal.pone.0291082 (PMC10861069; doi:10.1371/journal.pone.0291082)
Supplement: S1 Table — (DOCX) [file pone.0291082.s001.docx]

**S1 Table. Integrated SRH and HIV service utilisation data.**

| Facility type | NGO^1^ managed fixed model site | | NGO Partner managed fixed model site | | PPP^2^ site | | NGO managed mobile outreach | |
| --- | --- | --- | --- | --- | --- | --- | --- | --- |
| Service type | **Visits** | **%** | **Visits** | **%** | **Visits** | **%** | **Visits** | **%** |
| *HIV^3^ Testing and Counselling* | *41,177* | *83%* | *12,005* | *67%* | *412* | *7%* | *69,942* | *91%* |
| *TB^4^ screening & treatment* | *1,561* | *3%* | *1,922* | *11%* | *0* | *0%* | *0* | *0%* |
| *STI^5^ screening & treatment* | *557* | *1%* | *38* | *<1%* | *327* | *5%* | *39* | *<1%* |
| Cervical cancer *screening & cryotherapy* | *2,958* | *6%* | *88* | *<1%* | *256* | *4%* | *234* | *<1%* |
| *FP^6^* | *3,354* | *7%* | *3,881* | *22%* | *5,042* | *84%* | *7,063* | *9%* |
| Total | **49,607** |  | **17,934** |  | **6,037** |  | **77,278** |  |

^*^*^1^Non-governmental organization; ^2^Public private partnership; ^3^Human Immuno-Virus; ^4^Tuberculosis; ^5^Sexually transmitted infections; ^6^Family planning*
