# Supplementary material for: Provider and female client economic costs of integrated sexual and reproductive health and HIV services in Zimbabwe
Source: PLoS One. 2024 Feb 12;19(2):e0291082. doi: 10.1371/journal.pone.0291082 (PMC10861069; doi:10.1371/journal.pone.0291082)
Supplement: S2 Table — (DOCX) [file pone.0291082.s002.docx]

**S2 Table. Main reasons for health facility visit.**

| Reasons for your visit | Bambanani | Chitungwiza (NSC) | N.A.H^1^ | Total |
| --- | --- | --- | --- | --- |
|  | n (%) | n (%) | n (%) | N (%) |
| *HIV^3^ Testing and Counselling* | 59 (29.5%) | 36 (18.0%) | 45 (9.9%) | 140 (16.4%) |
| CD4^3^ cell count testing | 6 (3.0%) | 41 (20.5%) | 36 (7.9%) | 83 (9.7%) |
| TB^4^ results | 1 (0.5%) | 1 (0.5%) | 5 (1.1%) | 7 (0.8%) |
| FP^5^ services | 107 (53.5%) | 38 (19.0%) | 263 (57.7%) | 409 (47.6%) |
| Cervical cancer screening | 23 (11.5%) | 83 (41.5%) | 81 (17.8%) | 188 (21.9%) |
| Other | 4 (2.0%) | 1 (0.5%) | 26 (5.7%) | 31 (3.6%) |

^*^*^1^New Africa House; ^2^Human Immuno-Virus; ^3^Clusters of differentiation 4; ^4^Tuberculosis; ^5^Sexually transmitted infections; ^6^Family planning*
