# Supplementary material for: Provider and female client economic costs of integrated sexual and reproductive health and HIV services in Zimbabwe
Source: PLoS One. 2024 Feb 12;19(2):e0291082. doi: 10.1371/journal.pone.0291082 (PMC10861069; doi:10.1371/journal.pone.0291082)
Supplement: S3 Table — (DOCX) [file pone.0291082.s003.docx]

**S3 Table. Disease status and key characteristics of clients and their caregivers.**

| Variables | Levels | N (%) |
| --- | --- | --- |
| Participant’s HIV status | Negative  Positive  Prefer not to say  Don’t know | 595 (69.5%)  243 (28.4%)  4 (0.5%)  14 (16.4%) |
|  |  | 856 |
| Any caregiver | Yes  No | 151 (17.6%)  705 (82.4%) |
|  |  | 856 |
| Clients’ employment status | Unemployed  Unskilled  Semi-skilled  Student  Professional  Other | 318 (37.2%)  154 (18.0%)  103 (12.0%)  30 (3.5%)  79 (9.23%)  172 (20.1%) |
|  |  | 856 |
| Caregivers’ employment status | Unemployed  Unskilled  Semi-skilled  Student  Professional  Other | 63 (41.7%)  17 (11.3%)  17 (11.3%)  8 (5.3%)  27 (17.9%)  19 (12.6%) |
|  |  | 151 |
| Mode of transport | Public  Own/family  Other (walking, cycling) | 774 (90.4%)  22 (2.6%)  60 (7.0%) |
|  |  | 856 |
