# Supplementary material for: Provider and female client economic costs of integrated sexual and reproductive health and HIV services in Zimbabwe
Source: PLoS One. 2024 Feb 12;19(2):e0291082. doi: 10.1371/journal.pone.0291082 (PMC10861069; doi:10.1371/journal.pone.0291082)
Supplement: S4 Table — (DOCX) [file pone.0291082.s004.docx]

## **S4 Table. Breakdown of time spent accessing integrated SRH and HIV services.**

##

| Facility Site | Bambanani | | | Chitungwiza | | | N.A.H | | | Overall | | |
| --- | --- | --- | --- | --- | --- | --- | --- | --- | --- | --- | --- | --- |
|  | **Time (in hours)** | **Range (in hours)** | **Proportional contribution (%)** | **Time (in hours)** | **Range (in hours)** | **Proportional contribution (%)** | **Time (in hours)** | **Range (in hours)** | **Proportional contribution (%)** | **Time (in hours)** | **Range (in hours)** | **Proportional contribution (%)** |
| *Time spent travelling to facility: Mean (range)* | <1 | (<1-3) | 18% | <1 | (<1-5) | 16% | <1 | (<1-6) | 20% | 1 | (<1-6) | 18% |
| *Time spent at facility: Mean (range)* | 3 | (2-8) | 64% | 4 | (<1-8) | 68% | 3 | (2-8) | 59% | 3 | (<1-8) | 63% |
| *Time spent travelling home: Mean (range)* | <1 | (<1-3) | 18% | <1 | (<1-5) | 16% | <1 | (<1-6) | 20% | 1 | (<1-6) | 18% |
| Total | **5** |  | **100%** | **6** |  | **100%** | **5** |  | **100%** | **5** |  | **100%** |
