# Supplementary material for: Provider and female client economic costs of integrated sexual and reproductive health and HIV services in Zimbabwe
Source: PLoS One. 2024 Feb 12;19(2):e0291082. doi: 10.1371/journal.pone.0291082 (PMC10861069; doi:10.1371/journal.pone.0291082)
Supplement: S5 Table — (DOCX) [file pone.0291082.s005.docx]

### **S5a Table. Costs of integrated SRH and HIV services for several model sites (2015 US Dollars).**

|  | Mutare PPP NGO site | | | | | | | | | | | |  | Harare NGO site | | | | | | | | | | | |
| --- | --- | --- | --- | --- | --- | --- | --- | --- | --- | --- | --- | --- | --- | --- | --- | --- | --- | --- | --- | --- | --- | --- | --- | --- | --- |
|  | HTC | | STI screening and treatment | | TB screening | | Family planning services | | Cervical cancer screening | | All services | |  | HTC | | STI screening and treatment | | TB screening | | Family planning services | | Cervical cancer screening | | All services | |
|  | **US$** | **% of total** | **US$** | **% of total** | **US$** | **% of total** | **US$** | **% of total** | **US$** | **% of total** | **US$** | **% of total** |  | **US$** | **% of total** | **US$** | **% of total** | **US$** | **% of total** | **US$** | **% of total** | **US$** | **% of total** | **US$** | **% of total** |
| Capital costs |  |  |  |  |  |  |  |  |  |  |  |  | Capital costs |  |  |  |  |  |  |  |  |  |  |  |  |
| *Building space* | $119 | 0% | $84 | 2% | $119 | 1% | $118 | <1% | $122 | 1% | $560 | <1% | *Building space* | $1,824 | <1% | $226 | 2% | $428 | 5% | $933 | 1% | $308 | <1% | $3,719 | 1% |
| *Equipment costs* | $17,714 | 11% | $52 | 1% | $2,847 | 14% | $5,276 | 6% | $1,729 | 10% | $27,617 | 10% | *Equipment costs* | $34,673 | 7% | $1,062 | 9% | $1,598 | 18% | $2,195 | 1% | $3,268 | 5% | $42,796 | 6% |
| *Vehicles costs* | $668 | <1% | $2 | <1% | $107 | 1% | $216 | <1% | $5 | <1% | $997 | <1% | *Vehicles costs* | $1,265 | <1% | $17 | <1% | $48 | 1% | <1 | <1% | $91 | <1% | $1,524 | <1% |
| *Training costs* | $338 | <1% | $169 | 3% | $169 | 1% | $1,661 | 2% | $272 | 2% | $2,610 | 1% | *Training costs* | $338 | <1% | $169 | 1% | $169 | 2% | $441 | <1% | $272 | <1% | $1,390 | <1% |
| TOTAL CAPITAL | ***$18,838*** | ***12%*** | ***$307*** | ***6%*** | ***$3,241*** | ***16%*** | ***$7,271*** | ***9%*** | ***$2,127*** | ***12%*** | ***$31,785*** | ***11%*** | TOTAL CAPITAL | ***$38,100*** | ***8%*** | ***$1,475*** | ***12%*** | ***$2,243*** | ***25%*** | ***$3,673*** | ***2%*** | ***$3,939*** | ***6%*** | ***$49,429*** | ***7%*** |
| Recurrent costs |  |  |  |  |  |  |  |  |  |  |  |  | Recurrent costs |  |  |  |  |  |  |  |  |  |  |  |  |
| *Personnel* | $92,336 | 57% | $116 | 2% | $2,401 | 12% | $27,426 | 33% | $202 | 1% | $122,482 | 42% | *Personnel* | $316,720 | 67% | $3,249 | 26% | $2,401 | 27% | $119,213 | 75% | $9,138 | 13% | $450,721 | 62% |
| *Supplies* | $25,541 | 16% | $431 | 8% | $1,827 | 9% | $36,644 | 44% | $2,255 | 13% | $66,698 | 23% | *Supplies* | $64,158 | 14% | $2,076 | 17% | $1,098 | 12% | $29,641 | 19% | $52,209 | 76% | $149,183 | 21% |
| *Vehicle operation & maintenance* | $1,001 | 1% | $3 | <1% | $160 | 1% | $324 | <1% | $7 | <1% | $1,495 | 1% | *Vehicle operation & maintenance* | $1,882 | <1% | $25 | <1% | $95 | 1% | $153 | <1% | $135 | <1% | $2,292 | <1% |
| *Building operation & maintenance* | $426 | <1% | $302 | 6% | $426 | 2% | $423 | 1% | $437 | 3% | $2,014 | 1% | *Building operation & maintenance* | $988 | <1% | $122 | 1% | $232 | 3% | $505 | <1% | $167 | <1% | $2,014 | <1% |
| *Management & administrative costs* | $24,000 | 15% | $4,000 | 78% | $12,000 | 60% | $12,000 | 14% | $12,000 | 70% | $64,000 | 22% | *Management & administrative costs* | $52,615 | 11% | $5,538 | 44% | $2,769 | 31% | $5,538 | 3% | $3,360 | 5% | $69,822 | 10% |
| TOTAL RECURRENT | ***$119,304*** | ***88%*** | ***$852*** | ***94%*** | ***$4,815*** | ***84%*** | ***$64,817*** | ***91%*** | ***$2,902*** | ***88%*** | ***$ 256,689*** | ***89%*** | TOTAL RECURRENT | ***$383,747*** | ***92%*** | ***$5,473*** | ***88%*** | ***$3,826*** | ***75%*** | ***$149,513*** | ***98%*** | ***$61,649*** | ***94%*** | ***$674,031*** | ***93%*** |
| Total cost | **$138,142** |  | **$1,159** |  | **$8,056** |  | **$72,088** |  | **$5,029** |  | **$ 288,474** |  | Total cost | **$421,847** |  | **$6,948** |  | **$6,069** |  | **$153,186** |  | **$65,588** | **$23.31** | **$723,460** | 1% |
| Cost per stopover | $14 | | $137 | | $10 | | $22 | | $194 | | $16 | | Cost per stopover | $12 | | $22 | | $6 | | $47 | | $23 | | $15 | |
| Cost per HIV positive client | $87 | |  | |  | |  | |  | |  | | Cost per HIV positive client | $84 | |  | |  | |  | |  | |  | |
|  | NGO outreach | | | | | | | | | | | |  | Private-public-partnership | | | | | | | | | |  | |
|  | HTC | | STI screening and treatment | | TB screening | | Family planning services | | Cervical cancer screening | | All services | |  | HTC | | STI screening and treatment | | TB screening | | Family planning services | | Cervical cancer screening | | All services | |
| Capital costs |  |  |  |  |  |  |  |  |  |  |  |  | Capital costs |  |  |  |  |  |  |  |  |  |  |  |  |
| *Building space* | $0 | <1% | $0 | <1% | - | - | $0 | <1% | $0 | <1% | $ - | <1% | *Building space* | $140 | 2% | $45 | 1% | - | - | $45 | <1% | $45 | 1% | $275 | <1% |
| *Equipment costs* | $4,338 | 1% | $2 | <1% | - | - | $438 | <1% | $15 | <1% | $4,793 | <1% | *Equipment costs* | $127 | 2% | $100 | 3% | - | - | $868 | 1% | $46 | 1% | $1,140 | 1% |
| *Vehicles costs* | $12,416 | 2% | $7 | <1% | - | - | $1,254 | <1% | $42 | <1% | $13,718 | 1% | *Vehicles costs* | $0 | <1% | $0 | <1% | - | - | $0 | <1% | $0 | <1% | $ - | <1% |
| *Training costs* | $338 | <1% | $67 | 1% | - | - | $713 | <1% | $169 | 1% | $1,287 | <1% | *Training costs* | $338 | 4% | $169 | 6% |  |  | $713 | 1% | $169 | 3% | $1,559 | 2% |
| TOTAL CAPITAL | ***$17,093*** | ***2%*** | ***$76*** | ***1%*** | ***-*** | ***-*** | ***$2,405*** | ***1%*** | ***$225*** | **2%** | ***$19,799*** | ***2%*** | TOTAL CAPITAL | ***$605*** | ***8%*** | ***$314*** | ***10%*** | ***-*** | ***-*** | ***$1,626*** | ***2%*** | ***$260*** | ***4%*** | ***$2,974*** | ***3%*** |
| Recurrent costs |  |  |  |  |  |  |  |  |  |  |  |  | Recurrent costs |  |  |  |  |  |  |  |  |  |  |  |  |
| *Personnel* | $537,971 | 72% | $120 | 2% | - | - | $107,761 | 27% | $540 | 4% | $646,392 | 55% | *Personnel* | $3,169 | 40% | $1,006 | 33% | - | - | $50,243 | 61% | $591 | 9% | $55,009 | 54% |
| *Supplies* | $135,780 | 18% | $434 | 8% | - | - | $275,188 | 70% | $4,123 | 33% | $417,347 | 36% | *Supplies* | $564 | 7% | $1,145 | 37% | - | - | $30,392 | 37% | $4,511 | 69% | $36,611 | 36% |
| *Vehicle operation & maintenance* | $18,666 | 2% | $10 | 0% | - | - | $1,885 | 0% | $62 | <1% | $20,624 | 2% | *Vehicle operation & maintenance* | $0 | <1% | $0 | <1% | - | - | $0 | <1% | $0 | <1% | $ - | <1% |
| *Building operation & maintenance* | $0 | - | $0 | - | - | - | $0 | - | $0 | - | $ - | 0% | *Building operation & maintenance* | $0 | <1% | $0 | <1% | - | - | $0 | <1% | $0 | <1% | $ - | <1% |
| *Management & administrative costs* | $40,934 | 5% | $5,117 | 89% | - | - | $5,117 | 1% | $7,675 | 61% | $63,960 | 5% | *Management & administrative costs* | $3,639 | 46% | $607 | 20% | - | - | $607 | 1% | $1,213 | 18% | $6,672 | 7% |
| TOTAL RECURRENT | ***$733,351*** | ***98%*** | ***$5,682*** | ***99%*** | ***-*** | ***-*** | ***$384,834*** | ***99%*** | ***$4,725*** | **98%** | ***$1,148,322*** | ***98%*** | TOTAL RECURRENT | ***$3,733*** | ***92%*** | ***$2,151*** | ***90%*** | ***-*** | ***-*** | ***$80,635*** | ***98%*** | ***$5,101*** | ***96%*** | ***$98,292*** | ***97%*** |
| Total cost | **$750,443** |  | **$5,758** |  | **-** |  | **$387,238** |  | **$4,951** | **100%** | **$ 1,168,121** | **$750,443** | Total cost | **$4,338** |  | **$2,465** |  | **-** | **-** | **$82,261** |  | **$5,361** |  | **$101,266** |  |
| Cost per stopover | $11 | | $148 | |  | | $56 | | $54 | | $15 | | Cost per stopover | $19 | | $9 | |  | | $16 | | $26 | | $17 | |
| Cost per HIV positive client | $192 | |  | |  | |  | |  | |  | | Cost per HIV positive client |  | |  | |  | |  | |  | |  | |

|  | Chitungwiza NGO site | | | | | | | | | | | |
| --- | --- | --- | --- | --- | --- | --- | --- | --- | --- | --- | --- | --- |
|  | HTC | | STI screening and treatment | | TB screening | | Family planning services | | Cervical cancer screening | | All services | |
| Capital Costs |  |  |  |  |  |  |  |  |  |  |  |  |
| *Building space* | $610 | <1% | $430 | 5% | $610 | 5% | $490 | <1% | $430 | 3% | $ 2,570 | 1% |
| *Equipment costs* | $11,056 | 5% | $127 | 2% | $1,504 | 12% | $5,558 | 2% | $675 | 5% | $ 18,921 | 4% |
| *Vehicles costs* | $810 | <1% | $4 | <1% | $110 | 1% | $447 | <1% | $9 | <1% | $ 1,381 | <1% |
| *Training costs* | $169 | <1% | $169 | 2% | $169 | 1% | $985 | <1% | $272 | 2% | $ 1,764 | <1% |
| Total | ***$12,646*** | ***6%*** | ***$730*** | ***9%*** | ***$2,394*** | ***18%*** | ***$7,480*** | ***3%*** | ***$1,385*** | ***11%*** | ***$ 24,635*** | ***5%*** |
| Recurrent Costs |  |  |  |  |  |  |  |  |  |  |  |  |
| *Personnel* | $117,538 | 56% | $127 | 2% | $3,196 | 24% | $80,136 | 36% | $835 | 7% | $ 201,833 | 43% |
| *Supplies* | $32,353 | 16% | $573 | 7% | $519 | 4% | $125,907 | 56% | $3,489 | 28% | $ 162,842 | 35% |
| *Vehicle operation & maintenance* | $1,558 | 1% | $8 | <1% | $212 | 2% | $860 | <1% | $17 | <1% | $ 2,655 | 1% |
| *Building operation & maintenance* | $478 | <1% | $337 | 4% | $478 | 4% | $384 | <1% | $337 | 3% | $ 2,014 | <1% |
| *Management & administrative costs* | $43,826 | 21% | $6,261 | 78% | $6,261 | 48% | $9,391 | 4% | $6,261 | 51% | $ 72,000 | 15% |
| Total | ***$151,927*** | ***94%*** | ***$1,046*** | ***91%*** | ***$4,406*** | ***82%*** | ***$207,287*** | ***97%*** | ***$4,678*** | ***89%*** | ***$ 441,344*** | ***95%*** |
| Total costs | **$164,573** | **100%** | **$1,777** | **100%** | **$6,800** | **100%** | **$214,767** | **100%** | **$6,063** | **100%** | **$ 465,979** | **100%** |
| Cost per stopover | **$14** | | **$97.01** | | **$6.29** | | **$26.57** | | **$74.95** | |  | |
| Cost per diagnosis | $74 | |  | |  | |  | |  | | $ 17.89 | |

**S5b Table. Costs of integrated SRH and HIV services for several model sites (2015 US Dollars).**
