# Supplementary material for: Provider and female client economic costs of integrated sexual and reproductive health and HIV services in Zimbabwe
Source: PLoS One. 2024 Feb 12;19(2):e0291082. doi: 10.1371/journal.pone.0291082 (PMC10861069; doi:10.1371/journal.pone.0291082)
Supplement: S6 Table — (DOCX) [file pone.0291082.s006.docx]

**S6 Table. Workforce composition per site and department**

| **Site** | **Department** | **Services provided** | **Personnel type** | **Personnel numbers** |
| --- | --- | --- | --- | --- |
| **Chitungwiza NSC** | **TB Screening** | TB smear microscopy | Nurse midwife | 1 |
|  | **VIAC** | Cervical cancer screening & cryotherapy | Nurse midwife | 2 |
|  | **FP** | Group & individual counselling + FP | FP trained nurse | 2 |
|  | **STI screening** | Syndromic STI screening & treatment | STI trained nurse | 1 |
|  | **HTC** | Pre & post test counselling & testing | Nurse counselor | 4 |
|  |  |  |  |  |
| **Mutare NSC** | **TB Screening** | TB smear microscopy | Nurse midwife | 1 |
|  | **VIAC** | Cervical cancer screening & cryotherapy | Nurse midwife | 2 |
|  | **FP** | Group & individual counselling + FP | FP trained nurse | 2 |
|  | **STI screening** | Syndromic STI screening & treatment | STI trained nurse | 2 |
|  | **HTC** | Pre & post test counselling & testing | Nurse counselor | 3 |
|  |  |  |  |  |
| **NAH NSC** | **TB Screening** | TB smear microscopy | Nurse midwife | 1 |
|  | **VIAC** | Cervical cancer screening & cryotherapy | Nurse midwife | 2 |
|  | **FP** | Group & individual counselling + FP | FP trained nurse | 5 |
|  | **STI screening** | Syndromic STI screening & treatment | STI trained nurse | 2 |
|  | **HTC** | Pre & post test counselling & testing | Nurse counselor | 14 |
|  |  |  |  |  |
| **NAH Outreach** | **TB Screening** | TB smear microscopy | Nurse midwife | 1 |
|  | **VIAC** | Cervical cancer screening & cryotherapy | Nurse midwife | 1 |
|  | **FP** | Group & individual counselling + FP | FP trained nurse | 2 |
|  | **STI screening** | Syndromic STI screening & treatment | STI trained nurse | 1 |
|  | **HTC** | Pre & post test counselling & testing | FP trained nurse | 1 |
|  |  |  |  |  |
| **Chitungwiza Profam** | **TB Screening** | TB smear microscopy | Nurse midwife | 1 |
|  | **VIAC** | Cervical cancer screening & cryotherapy | Nurse midwife | 1 |
|  | **FP** | Group & individual counselling + FP | FP trained nurse | 2 |
|  | **STI screening** | Syndromic STI screening & treatment | STI trained nurse | 1 |
|  | **HTC** | Pre & post test counselling & testing | Nurse counselor | 1 |
|  |  |  |  |  |
