# Supplementary material for: Provider and female client economic costs of integrated sexual and reproductive health and HIV services in Zimbabwe
Source: PLoS One. 2024 Feb 12;19(2):e0291082. doi: 10.1371/journal.pone.0291082 (PMC10861069; doi:10.1371/journal.pone.0291082)
Supplement: S7 Table — (DOCX) [file pone.0291082.s007.docx]

**S7 Table. Space measurements per site**

| **Chitungwiza NSC** | | | | **Mutare NSC** | | | | NAH NSC | | | | **Chitungwiza Profam Site** | | | |
| --- | --- | --- | --- | --- | --- | --- | --- | --- | --- | --- | --- | --- | --- | --- | --- |
| **Building (Property owned by Old Mutual)** | **Area (sq metres)** | | | **Building (Property owned by Red Cross)** | **Area (sq metres)** | | | **Building (Property owned by Old Mutual)** | **Area (sq metres)** | | | **Building (Property owned by Chitungwiza Hosp)** | **Area (sq metres)** | | |
|  |  |  |  |  |  |  |  |  |  |  |  |  |  |  |  |
|  |  |  |  |  |  |  |  |  |  |  |  |  |  |  |  |
| **SRH** | **Length m²** | **Width m²** | **Total area m²** | **SRH** | **Length m²** | **Width m²** | **Total area m²** | **SRH** | **Length m²** | **Width m²** | **Total area m²** | **SRH** | **Length m²** | **Width m²** | **Total area m²** |
| Jardel room 1 | 4 | 3 | 12 | FP room | 2.5 | 4.5 | 11.25 | SRH Managers Office | 4 | 4 | 16 | Profam room | 3 | 3 | 9 |
| Cervical cancer room 2 | 4 | 3 | 12 | VIAC room | 2.5 | 5 | 12.5 | FP room | 5 | 4 | 20 |  |  |  |  |
| Profam room 3 (Short term methods) | 4 | 3 | 12 | VIAC & FP waiting area | 7.5 | 1.5 | 11.25 | Pharmacy | 3 | 4 | 12 |  |  |  |  |
| STI screening | 4 | 3 | 12 | **Total sq metres** |  |  | **35** | Doctors office | 3 | 3 | 9 |  |  |  |  |
|  |  |  |  |  |  |  |  | VIAC clinic | 4 | 6 | 24 |  |  |  |  |
|  |  |  |  | **ART** |  |  |  | SRH/FP waiting area | 6 | 4 | 24 |  |  |  |  |
|  |  |  |  | ART Reception | 4 | 6.5 | 26 | SRH/FP reception area | 6 | 4 | 24 |  |  |  |  |
|  |  |  |  | ART Consultation rooms |  |  |  | Counselling rooms 1 | 3 | 4 | 12 |  |  |  |  |
|  |  |  |  | 1 | 3.5 | 3.5 | 12.25 | Counselling rooms 2 | 3 | 4 | 12 |  |  |  |  |
|  |  |  |  | 2 | 2.5 | 2.5 | 6.25 | Counselling rooms 3 | 4 | 3 | 12 |  |  |  |  |
|  |  |  |  | 3 | 2.5 | 3.5 | 8.75 | Counselling rooms 4 | 3 | 3 | 9 |  |  |  |  |
|  |  |  |  | ART Pharmacy | 2.5 | 3 | 7.5 | Counselling rooms 5 | 3 | 4 | 12 |  |  |  |  |
|  |  |  |  | **Total sq metres** |  |  | **60.75** | TT/STI room | 2.5 | 3 | 7.5 |  |  |  |  |
| **Total sq metres** |  |  | **48** | **Total sq metres** |  |  | **95.75** | **Total sq metres** |  |  | **193.5** | **Total sq metres** |  |  | **9** |
| **HTC** |  |  |  | **HTC** |  |  |  | **HTC** |  |  |  | **HTC** |  |  |  |
| HTC room 4 | 4 | 3 | 12 | Counselling room 1 | 3 | 3 | 9 | HTC Managers office | 4 | 4 | 16 | Counselling room 1 | 4 | 4 | 16 |
| HTC room 5 | 4 | 3 | 12 | Counselling room 2 | 3 | 2.5 | 7.5 | HTC waiting area | 6 | 4 | 24 | Counselling room 2 | 4 | 3 | 12 |
| HTC room 7 | 4 | 3 | 12 | Counselling room 3 | 2.5 | 2.5 | 6.25 | HTC reception | 2.5 | 2.5 | 6.25 |  |  |  |  |
| HTC room 9 | 4 | 3 | 12 |  |  |  |  | Foyer waiting area | 6 | 4 | 24 |  |  |  |  |
|  |  |  |  |  |  |  |  | Waiting area 1 | 4 | 4 | 16 |  |  |  |  |
|  |  |  |  |  |  |  |  | Screening room 1 | 3 | 2.5 | 7.5 |  |  |  |  |
|  |  |  |  |  |  |  |  | Screening room 2 | 4 | 4 | 16 |  |  |  |  |
|  |  |  |  |  |  |  |  | Counselor In Charges office | 5.5 | 4.5 | 24.75 |  |  |  |  |
|  |  |  |  |  |  |  |  | Counselling room 1 | 3 | 4 | 12 |  |  |  |  |
|  |  |  |  |  |  |  |  | Counselling room 2 | 5 | 2 | 10 |  |  |  |  |
|  |  |  |  |  |  |  |  | Counselling room 3 | 3.5 | 3.5 | 12.25 |  |  |  |  |
|  |  |  |  |  |  |  |  | Counselling room 4 | 3.5 | 3.3 | 11.55 |  |  |  |  |
|  |  |  |  |  |  |  |  | Counselling room 5 | 3.5 | 1.5 | 5.25 |  |  |  |  |
|  |  |  |  |  |  |  |  | Counselling room 6 | 5 | 4.5 | 22.5 |  |  |  |  |
|  |  |  |  |  |  |  |  | Counselling room 7 | 4 | 6 | 24 |  |  |  |  |
|  |  |  |  |  |  |  |  | Sputum collection area | 4 | 6 | 24 |  |  |  |  |
|  |  |  |  |  |  |  |  | Counselling room 21 | 3 | 4 | 12 |  |  |  |  |
|  |  |  |  |  |  |  |  | Counselling room 22 | 3 | 3 | 9 |  |  |  |  |
|  |  |  |  |  |  |  |  | Counselling room 29 | 4 | 3 | 12 |  |  |  |  |
|  |  |  |  |  |  |  |  | Waiting room 2 | 4 | 8 | 32 |  |  |  |  |
|  |  |  |  |  |  |  |  | Cabinet area | 3 | 2 | 6 |  |  |  |  |
| **Total sq metres** |  |  | 48 | **Total sq metres** |  |  | **22.75** | **Total sq metres** |  |  | **327.05** | **Total sq metres** |  |  | **28** |
| **Shared facilities** |  |  |  | **Shared facilities** |  |  |  | **Shared facilities** |  |  |  | **Shared facilities** |  |  |  |
| Managers office | 5 | 3 | 15 | HTC & SRH reception | 6 | 3.5 | 21 | Main lab | 4 | 6 | 24 |  |  |  |  |
| Waiting area | 6 | 16 | 96 | HTC lab | 2 | 2.5 | 5 | Routine lab | 4 | 5 | 20 |  |  |  |  |
| Screening room (TB, HIV, CD4 count) | 4 | 3 | 12 | Lab waiting area | 3.5 | 1.5 | 5.25 | TB lab | 3 | 4 | 12 |  |  |  |  |
| Store room | 4 | 3 | 12 | Waiting area | 6 | 6 | 36 | CD4 lab | 2.5 | 3 | 7.5 |  |  |  |  |
| Kitchen/kitchen | 5 | 5 | 25 | Kitchen | 5 | 2.5 | 12.5 | Shared reception | 4 | 6 | 24 |  |  |  |  |
| Backyard | 13 | 14 | 182 | Toilet | 2 | 2 | 4 | Kitchen | 5 | 4 | 20 |  |  |  |  |
| Corridor | 13 | 2 | 26 |  |  |  |  | Functions room | 3.5 | 10 | 35 |  |  |  |  |
|  |  |  |  |  |  |  |  | Filing room | 3 | 4.5 | 13.5 |  |  |  |  |
|  |  |  |  |  |  |  |  | Clients toilets | 4 | 3 | 12 |  |  |  |  |
|  |  |  |  |  |  |  |  | Staff ladies toilets | 5 | 1 | 5 |  |  |  |  |
|  |  |  |  |  |  |  |  |  | 3 | 2.5 | 7.5 |  |  |  |  |
|  |  |  |  |  |  |  |  |  | 2.5 | 1.5 | 3.75 |  |  |  |  |
|  |  |  |  |  |  |  |  | Male toilets | 4 | 1 | 4 |  |  |  |  |
