# Supplementary material for: Provider and female client economic costs of integrated sexual and reproductive health and HIV services in Zimbabwe
Source: PLoS One. 2024 Feb 12;19(2):e0291082. doi: 10.1371/journal.pone.0291082 (PMC10861069; doi:10.1371/journal.pone.0291082)
Supplement: S8 Table — (DOCX) [file pone.0291082.s008.docx]

**S8 Table. Minimum Wage in Zimbabwe for the mining, printing and packaging, ceramic, insurance, transport industry.**

| **Industrial sector** | **Monthly Wage (US)** |
| --- | --- |
| Mining | 100 |
| Civil Service | 150 |
| Agriculture | 42.5 |
| Engineering | 150 |
| Commercial | 150 |
| Food Federation | 125 |
| Clothing | 80 |
| PTUZ | 80 |
| Railway Artisans | 391 |
| Catering | 85 |
| Chemicals | 105 |
| Domestics | 30 |
| Iron and Steel | 90 |
| Furniture | 45 |
| Leather | 138 |
| Graphical | 120 |
| Pulp and Paper | 155 |
| Mission Hospitals | 100 |
| Textile | 90 |
| Media | 250 |
| Railway Engine Man | 120 |
| Security Guard | 150 |
| Tobacco | 190 |
| Rural District Council | 70 |
| Urban Council | 70 |
| Telone Comm. | 136 |
| Transport | 180 |
| Cement and Lime | 150 |

* Current minimum wages for various sectors in Zimbabwe in United States dollars courtesy of the Zimbabwe Congress of Trade Union (ZCTU). The annual inflation rate is at 2.2% while the Poverty Datum line stands at US$500. Adapted from the [Africa Labour, Research and Education Institute , Lome](https://www.wageindicator.org/), <https://alrei.org/research/data-bases/minimum-wages/zimbabwe> and https://www.wageindicator.org/
