# Supplementary material for: Provider and female client economic costs of integrated sexual and reproductive health and HIV services in Zimbabwe
Source: PLoS One. 2024 Feb 12;19(2):e0291082. doi: 10.1371/journal.pone.0291082 (PMC10861069; doi:10.1371/journal.pone.0291082)
Supplement: S1 File — (DOCX) [file pone.0291082.s009.docx]

**S 1 File. Costing overview**

**Introduction**

In this cost analysis we take the provider's perspective and calculate costs in 2015 United States Dollars (US$). We did not need to convert currencies as United States dollars were the principal currency in use in Zimbabwe at the time following the demise of the local currency earlier in 2009 due to hyper-inflation.

**Costing methods**

Annual costs were collected retrospectively. We assumed there was no difference between delivery of integrated SRH and HIV services during the data collection period compared to the prior 12 months.

**Facility observations**

Upon arrival at each facility site we first conducted mapping and studied client flow patterns and then proceeded to document how resources were combined to produce integrated SRH and HIV services. We collected SRH and HIV service utilization data (production figures) retrospectively for each month from registers maintained in each unit or service department and monthly/quarterly reports. We also accessed electronic records from the program M&E department which helped in instances of missing numbers. We also observed provision of integrated services through time and motion analysis (the gold standard tool for measuring staff time allocation using direct observation).

For each facility we recorded type of model, size and location, facility characteristics, operational details, staffing levels and types, and client volumes. We collected data on types and number of HIV tests administered, total HIV cases diagnosed, number of STI screenings and cases identified, and number of TB screenings with smear microscopy as well as cases identified. We also collected number of cervical cancer screenings and treatments for abnormalities performed using Visual Inspection with Acetic Acid and Cervicography (VIAC) and cryotherapy.

**Detailed cost data collection**

We quantified each ingredient or resource component required to provide individual SRH and HIV services and multiplied it by its price to derive its total cost contribution to unit cost. Overhead financial expenditures (costs of resources shared across different SRH and HIV services) were allocated in a step wise fashion starting with central office, site and then to their final cost centres (SRH and HIV services).

We included both actual financial expenditures on integrated SRH and HIV services and economic costs (the value of all resources including those for which there were no financial expenditures such as donated goods, and services incurred elsewhere). Any donated goods were valued at their opportunity costs determined using National Pharmaceutical Company (NatPharm) prices. Costs were split into capital (building space, equipment, vehicle purchase costs, and training) and recurrent costs (staff salaries, drugs, diagnostics, and supplies costs, vehicle operation and maintenance costs, and building operation [utility] and maintenance costs). Capital costs are generally considered to have a lifespan of greater than one year and to cost more than $100 per unit.

**Capital costs - Building space, furniture, equipment, and vehicles.**

Facility and departmental space were physically measured using tape measures (in square metres) and the space estimates valued using rental estimates from a mid-year property market survey conducted by a major real estate company. All furniture and equipment listed behind every room door, and vehicles on each sites inventory that contributed to delivery of integrated SRH and HIV services were physically verified, recorded, and then valued using lowest procurement price estimates from the NGO finance department. Equipment found in each standalone room such as STI or FP was allocated to the specific integrated SRH and HIV service provided there 100%. Purchase prices of equipment and vehicles were annualized then summed up and divided by the annual number of services provided for the respective integrated service. For this purpose equipment and vehicles were assigned a life expectancy of between 3-7 years and 7 to 10 years for furniture.

**Capital costs - Training costs.**

Details on staff training workshops provided to enable staff to provide integrated SRH and HIV services were gathered through feedback from facility managers, departmental head or staff. Training costs include facilitator and participant time, catering costs, and training materials. For each service we summed up staff training costs (assuming an amortization period of 4 years) and divided them by the total number of services provided in that department to derive the per service training cost.

**Recurrent costs**

Recurrent costs included personnel salaries, drugs and supplies, vehicle operation and maintenance, and building operation (utility) and maintenance. We allocated utilities, building rentals and maintenance charges using room space whereas management and administrative costs were allocated based on the total number of staff members working in each unit or integrated service department.

**Recurrent costs - Personnel time and costs**

As a basis for costing time spent providing integrated SRH and HIV services we collected data through facility manager’s, departmental supervisors, and staff. We also conducted time and motion analysis to track and breakdown the time taken by integrated service staff in providing integrated SRH and HIV services. In-order to ensure confidentiality for clients, and given the sensitive nature of individual SRH and HIV services time and motion observations were conducted from outside consultation rooms. We divided the annual salary rate (monthly salary rate x 12 months) of staff providing individual services by the total annual number of hours worked (assuming 40 hour week x 48 weeks per year) to derive their hourly salary rate. We then summed up the total time spent by staff providing their respective services and multiplied their time contribution to an integrated SRH and HTC service by their hourly salary rate.

**Drugs and supplies**

Costs of drugs and supplies were allocated based on actual resource usage obtained through analysis of stock card records, inventory lists as well as records of delivered supplies obtained from facility sites. We accessed unit prices of consumables from NGO and the National Pharmaceutical Company (NatPharm) of Zimbabwe. NatPharm is the appointed agent responsible for bulk procurement, storage and distribution of medical supplies to public health institutions. We multiplied the total units of each supply item used by its price per unit to produce the total cost for that item. Total costs of all supply items were summed up and divided by the total number of each integrated service provided to derive the cost contribution of supplies.

**Utilities, vehicle maintenance, and external services**

For each facility site, we accessed building utilities data (electricity, water and communications), vehicle operation, and maintenance and costs of external services such as waste management from the finance department.
