# Supplementary material for: Provider and female client economic costs of integrated sexual and reproductive health and HIV services in Zimbabwe
Source: PLoS One. 2024 Feb 12;19(2):e0291082. doi: 10.1371/journal.pone.0291082 (PMC10861069; doi:10.1371/journal.pone.0291082)
Supplement: S2 File — (DOCX) [file pone.0291082.s010.docx]

**S 2 File. SRH and HIV integration client exit questionnaire**

**An evaluation of sexual and reproductive health services**

**provided by PSI Zimbabwe across different health care delivery sites in Zimbabwe.**

**EXIT QUESTIONNAIRE**

I will ask you a few questions about the visit you made to the New Start Centre today. This should take less than five minutes to complete. Please feel free to give me your honest responses. We are not asking for your name or any information that can be used to identify you. It will therefore not be possible for anyone to link your responses to you. Some of the questions will ask about which services you were offered or chose to access. In addition we will ask you questions about how much time you have spent at the clinic and any costs you have incurred as a result. You do not need to answer any question that makes you feel uncomfortable or that you do not want to answer.

1. What is the main reason you visited the clinic today?
   1. HIV testing and counselling
   2. CD4 cell count testing
   3. To collect TB results
   4. To access family planning services
   5. To access cervical cancer screening services
   6. Other (specify): ______________________
2. Did you access the services you came for?
   1. Yes
   2. No
3. How many times did you have to visit the clinic in order to access this service? (do not count attending repeatedly for the same service but count the number of times you attended to access the services you accessed today).
   1. Once (I only came today)
   2. Twice
   3. Three times
   4. Four times
   5. More than four times
4. On top of the services you originally came to access today, did New Start Centre staff recommend other services?
   1. Yes
   2. No

***If yes***

1. What other services were recommended?***Mark all that apply***
   1. HIV testing and counselling
   2. CD4 cell count testing
   3. HIV care and treatment
   4. TB testing
   5. Family planning services
   6. Cervical cancer screening services
   7. Other (specify): ______________________

***If family planning services were recommended***

1. What methods of family planning were offered? ***Mark all that apply***
   1. Pills
   2. Depo injection
   3. Jadelle
   4. IUCD (loop)
2. Did you take up the recommended services?
   1. Yes
   2. No
   3. Not yet, but I am planning to make another visit to take up the services
3. What is your main occupation?
   1. If employed what are your earnings in US$?
4. What mode of transport did you use to get to the clinic today?
   1. Public transport
   2. Own or family transport
   3. Other (specify): ________________
5. How long did it take you to get to the clinic facility? (one way)

_______Hours walking ________hours on public transport __________Other

1. How much was your transport cost to get to the clinic today? ______________
2. How much money will it take to get back home today? __________
3. How much time in minutes did you spend at the clinic today from the time you arrived to the time you are leaving? ______________
4. Would you say this visit cost you any additional money aside from that which you used for transport?
   1. Yes
   2. No

If yes, describe: _________________________________

1. Did anyone accompany you to the clinic today?
   1. Yes
   2. No
2. If yes why did they accompany you? ____________________________
3. What is their main occupation? ________________________________
4. If employed what are their earnings in US$?______________________
5. What is your HIV status?
   1. Negative
   2. Positive
   3. Prefer not to say
   4. I don’t know
